# Role of STING in the treatment of non-small cell lung cancer

**DOI:** 10.1186/s12964-024-01586-x

**Published:** 2024-04-02

**Authors:** Wenhua Tang, Wenjie Zhou, Mei Ji, Xin Yang

**Affiliations:** Departments of Oncology, The Third Affiliated Hospital of Soochow University, 185 Juqian Street, Changzhou, 213003 China

**Keywords:** cGAS-STING, Non-small cell lung cancer, Immunotherapy, Immune checkpoint inhibitors, Nanoparticles

## Abstract

Non-small cell lung cancer (NSCLC) is a prevalent form of lung cancer. Patients with advanced NSCLC are currently being treated with various therapies, including traditional radiotherapy, chemotherapy, molecular targeted therapies and immunotherapy. However, a considerable proportion of advance patients who cannot benefit from them. Consequently, it is essential to identify a novel research target that offers an encouraging perspective. The stimulator of interferon genes (STING) has emerged as such a target. At present, it is confirmed that activating STING in NSCLC tumor cells can impede the proliferation and metastasis of dormant tumor cells. This review focuses on the role of STING in NSCLC treatment and the factors influencing its activation. Additionally, it explores the correlation between STING activation and diverse therapy modalities for NSCLC, such as radiotherapy, chemotherapy, molecular targeted therapies and immunotherapy. Furthermore, it proposes the prospect of innovative therapy methods involving nanoparticles, with the aim of using the features of STING to develop more strategies for NSCLC therapy.

## Introduction

Lung cancer is one of the most prevalent cancers worldwide, with one of the highest mortality rates [1]. According to histological classification, it is essentially divided into non-small cell lung cancer (NSCLC) and small cell lung cancer (SCLC), with 80–85% of all lung cancer patients having NSCLC [2]. Currently, various approaches are employed for treating NSCLC, including chemotherapy, radiotherapy, molecular targeted therapy, and immunotherapy. Among these, immunotherapy is a prominent area of research, garnering considerable attention because of its therapeutic potential. Immune checkpoint blockade (ICB) therapy has shown significant benefits for treating NSCLC by enhancing the immune system of patients to combat the disease. ICB therapy enhances anti-tumor immunity by blocking intrinsic immune factors that are down-regulated, such as cytotoxic T lymphocyte antigen 4 (CTLA-4), programmed cell death receptor 1 (PD-1), or its ligand, programmed cell death ligand 1 (PD-L1) [3]. However, a considerable proportion of patients diagnosed with advanced lung cancer continue to be unresponsive to immunotherapy, and the reported response rates for anti-PD immunotherapy in NSCLC are only about 20% [4]. Additionally, another common limitation of existing treatments is that most advanced patients develop resistance to anti-tumor drugs used in various traditional approaches to treating NSCLC, rendering first-line therapies unsuitable for this population. As a result, it is essential for us to find new therapeutic targets to overcome traditional treatments resistance.

Stimulator of Interferon Genes (STING), encoded by TMEM173, plays an important role in cytoplasmic DNA sensing [5]. Its widespread expression in patients with NSCLC correlates with T cell function genes, adenocarcinoma histology, EGFR or KRAS mutations, and improved overall survival [6]. Consequently, STING has emerged as a promising immunotherapeutic target for NSCLC. Currently, the inhibition of PD-L1 is associated with the senescence of cancer cells. Evidence supports that depleting PD-L1 inhibits tumor growth by inducing cell senescence through STING upregulation [7]. Furthermore, activation of the intracellular STING protein drives various immune responses, leading to immune-mediated tumor elimination, anti-tumor immune memory generation, and modification of the tumor microenvironment (TME) [8]. In mouse models, ICB therapy eliminates tumor cells by T cell reactivation in the TME, dendritic cell authorization for T cell initiation and clonal expansion, and NK cell activation [9]. ‘Cold’ (“immune-desert”) tumors can be converted to ‘hot’ (“immunoinflammatory”) tumors with increased immune cell infiltration by remodeling the TME through activation of the STING pathway [10]. Reports indicate that the anti-tumor innate immune response interacts with the expression of other immune markers through the STING pathway, suggesting a potential therapy for recurrent NSCLC [11]. Nonetheless, it should be noted that STING also exerts a negative regulatory role in tumor cells, mediating immune resistance and fostering tumor growth. Persistent activation of STING signaling in tumors can cause cancer progression by tipping the balance in favor of an immunosuppressive TME [12]. Miao et al. found STING was more highly expressed in cancer lesions than in normal tissues and high expression of STING was associated with poorer survival of patients [13]. Additionally, existing research indicates that STING plays an important role in facilitating polyaromatic hydrocarbon 7,12-dimethylbenz[α]anthracene (DMBA)-induced tumorigenesis [14]. The aberrant activation of STING can disrupt immune homeostasis, contributing to pathological conditions and human diseases [15]. However, STING has been observed to be beneficial in most NSCLC treatments due to its ability to inhibit tumor growth. By investigating suitable approaches to activate the STING pathway, it is possible to widely activate STING in chemotherapy, radiotherapy, molecularly targeted therapies, and immunotherapy for NSCLC therapy with its strong anti-tumor effect. Despite the existence of certain challenges that need to be addressed like targeting STING in cancer cells, the research about the role of STING in NSCLC treatment is still beneficial for improving the NSCLC patients’ survival rate. This review mainly focuses on the role of STING in treating NSCLC and the factors affecting STING activation. Additionally, a concise overview of a novel therapy involving the combination of STING and nanoparticles is provided, aiming to offer additional strategies for treating NSCLC.

## STING plays a dual regulatory role in NSCLC tumor cells

### STING plays a role in promoting tumor proliferation for treating NSCLC

#### STING plays a role in promoting the growth of tumor characterized by low antigenicity via indoleamine 2,3-dioxygenase (IDO) activation

**Fig. 1:**
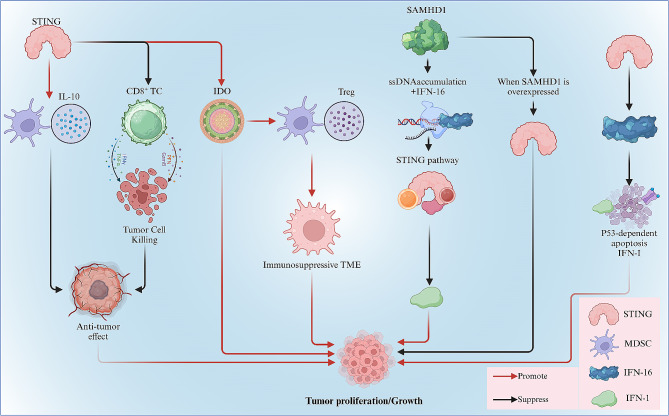
In the LLC model, STING can reduce CD8^+^ T cell infiltration and tumor cell killing, while increasing MDSC infiltration and IL-10 production in the TME. Furthermore, STING can induce local IDO activity to promote LLC growth in low antigenicity TME. SAMHD1 overexpression can negatively regulate STING in lung cancer and inhibit tumor proliferation. In addition, STING-mediated IFI16 degradation can negatively regulate IFI16-mediated p53-dependent apoptosis and downstream IFN-I production in NSCLC, which can promote tumor proliferation

Tumor antigenicity affects the DNA immune response in TME, which is a key factor affecting the dependence on STING to induce indoleamine 2,3-dioxygenase (IDO) and promote Lewis lung cancer (LLC) growth. In the LLC model, STING reduced CD8^+^ T cell infiltration and tumor cell killing while increasing myeloid-derived suppressor cells (MDSCs) infiltration and IL-10 production in the TME. Lemous et al. observed that compared to B6 (WT) mice, the tumor of the STING-deficient (STING-KO) mice with B6 backgrounds is smaller after 20 days. Additionally, compared to naive WT mice, IDO activity was elevated significantly in tumor-draining lymph nodes (TDLNs) from B6 (WT) mice. In contrast, TDLN IDO activity was not induced in mice lacking STING or IFN-I receptor (IFNAR) genes. Through relative experiments, Lemous et al. demonstrated that STING / IFN-I signal transduction is activated by cytoplasmic DNA sensing in low antigenicity TME to induce local 2,3-dioxygenase (IDO) activity and other tolerogenic responses that promote LLC growth [16]. Moreover, immunosuppressive TME promotes tumor cell growth, and tumor-infiltrating regulatory T cells (Tregs) and MDSCs are key factors for maintaining this immunosuppression [17]. Horvath et al. have indicated that the poor efficacy of tumor immunotherapy is related to the infiltration of Tregs and MDSCs [18], whereas IDO is a critical promoter of Treg and MDSC proliferation/activation [19]. The enhanced IDO activity induced by STING activation may be associated with the formation of an immunosuppressive microenvironment within the tumor, which makes tumor immunotherapy ineffective and promotes tumor cell growth. In summary, diverse tumor antigenicity results in distinct roles for STING within tumor cells. Specifically, the activation of STING enhances IDO activity during the growth of low antigenicity tumors, thereby contributing to the promotion of tumor growth (Fig. 1).

#### STING plays a negative regulatory role in NSCLC immunotherapy in the case of SAMHD1 overexpression

Sterile alpha motif domain and histidine-aspartate domain-containing protein 1(SAMHD1) is a deoxynucleotide triphosphate (dNTP) triphosphate hydrolase that manages cell proliferation and apoptosis by regulating intracellular dNTP levels or DNA damage responses [20]. Shang et al. have reported that higher serum levels of SAMHD1 are associated with NSCLC progression, and patients with NSCLC have significantly elevated serum levels of SAMHD1 [21]. SAMHD1 expression inhibits the accumulation of single-stranded DNA (ssDNA) in the cytoplasm, which is accompanied by a downregulation of the DNA sensor IFN-γ-inducible protein 16 (IFI16). It also hinders the activation of the STING pathway, which can inhibit the expression of IFN1 and weaken anti-tumor immunity in NSCLC treatment [22, 23].However, Wang et al. have revealed that the overexpression of SAMHD1 inhibits the proliferation of lung cancer cells [24]. Wu et al. transduced the human STING/SAMHD1 gene stably into the NSCLC cell lines with a STING/SAMHD1 expressing lentivirus and both of their expression levels were determined by RT-qPCR and western blot. Subsequently, it is indicated that the overexpression of SAMHD1 can suppress the progression of lung adenocarcinoma (LUAD) by inhibiting the expression of STING and STING can be used as a potential downstream target of SAMHD1 in lung cancer after MTT assays and Transwell assays [25]. And combined with the previous finding made by Coquel et al. that SAMHD1 can block the STING pathway by preventing accumulation of cytosolic ssDNA and induction of IFN-I [23], it is tempting to speculate that STING overexpression may diminish the inhibitory effect of SAMHD1 on tumor cells. However, the exact mechanism of this action remains unknown. Currently, it is confirmed that when SAMHD1 is overexpressed, the STING pathway becomes negatively regulated in the context of immunotherapy for NSCLC(Fig. 1).

#### STING affects p53-dependent apoptosis by degrading IFI16 to promote the proliferation of cancer cells

IFI16 is a member of the PYHIN protein family, which contains a pyrin domain and two DNA-binding HIN domains. It can be used as an intracellular DNA sensor that facilitates the induction of interferon-β (IFNβ). STING is a key mediator of IFNβ response to DNA and is recruited to IFI16 in response to DNA stimulation [26]. IFI16 can stimulate downstream IFN-I production and antiviral immunity. Furthermore, STING-mediated IFI16 degradation serves as a mechanism to negatively regulate IFN-I induced by viral DNA, thereby preventing excessive antiviral immunity [27]. The p53 tumor suppressor encoded by the TP53 gene is frequently mutated or functionally inactivated in a variety of human cancers. The p53 protein also acts as a tumor suppressor by inhibiting cell cycle progression and inducing apoptosis [28]. Through flow cytometry apoptosis detection and immunoblot assays, Li et al. found that IFI16 and nutlin-3, a p53 pathway activator, synergistically induce apoptosis in NSCLC cells [29]. Protein kinase-R have been shown to be responsible for p53 serine 392 phosphorylation [30], which is critical for the IFI16-p53-dependent apoptosis. However, overexpression of STING suppresses p53 serine 392 phosphorylation, p53 transcriptional activity, expression of p53 target genes, and p53-dependent mitochondrial depolarization and apoptosis. It is indicate that STING-mediated IFI16 degradation can negatively regulate IFI16-mediated p53-dependent apoptosis in NSCLC, suggesting that STING has a tumor-promoting effect in some cancer types due to its potent ability to degrade IFI16 upstream [29]. In summary, STING-mediated degradation of IFI16 impacts p53-mediated cell cycle arrest, ultimately leading to tumor cell proliferation (Fig. 1).

### STING plays a role in inhibiting tumor proliferation in treating NSCLC

#### Cancer cell-intrinsic STING activation suppresses LUAD metastasis by promoting infiltration of NK cells and T cells

Local recurrence and metastasis of cancer cells are the main causes of death in patients with lung cancer. In fact, the absolute risk of metastasis surpasses that of local recurrence, and metastasis indicates a notably high mortality rate throughout all stages, including the early stage of lung cancer detection [31]. Currently, it is well established that the immune surveillance of NK cells can inhibit the proliferation of cancer cells, inducing a stationary state. Conversely, T cell-depleted tumor cells exhibit invasive capabilities and the ability to disseminate [32, 33]. Therefore, it is critical to promote the infiltration of NK and T cells to inhibit the spread of cancer cells. According to the study made by Hu et al., they used models of indolent lung adenocarcinoma metastasis to identify cancer cell-intrinsic determinants of immune reactivity during exit from dormancy. After genetic screens of tumor-intrinsic immune regulators, they identified the STING pathway as a suppressor of metastatic outbreak. Additionally, immunofluorescence analysis of NK cells (anti-NKp46 staining) and T cells (anti-CD3 staining) in Mouse lung cancer cell line KP-482T1 metastases showed an increased proportion of infiltrated NK and T cells in response to STING overexpression, which indicated that the activation of STING could suppresses LUAD metastasis by promoting infiltration of NK and T cells [34]. Furthermore, Lohinai et al. analyzed TCGA data of STING in adenocarcinoma tissue microarrays and the result showed that low expression of STING in adenocarcinoma, correlates with poor survival. Further TCGA analysis showed STING expression correlates positively with T cell function and development genes in lung cancer, while STING expression negatively correlates with common tumor proliferation genes [6]. Besides, An et al. have found that negative correlation between the expression level of STING and its methylation level in NSCLC cells [35]. Conversely, a positive correlation has been observed between the level of STING methylation and poor prognosis in lung cancer [36]. It is reasonable to speculate that the expression of STING in cancer cell could lead to good prognosis for patients. In summary, the expression of STING showed a strong negative correlation with tumor proliferation markers, supporting STING’s role as an immune promoter and tumor suppressor [6].

#### STING reprograms the TME by activating the cGAS-STING pathway and inducing IFN-I production

**Fig. 2:**
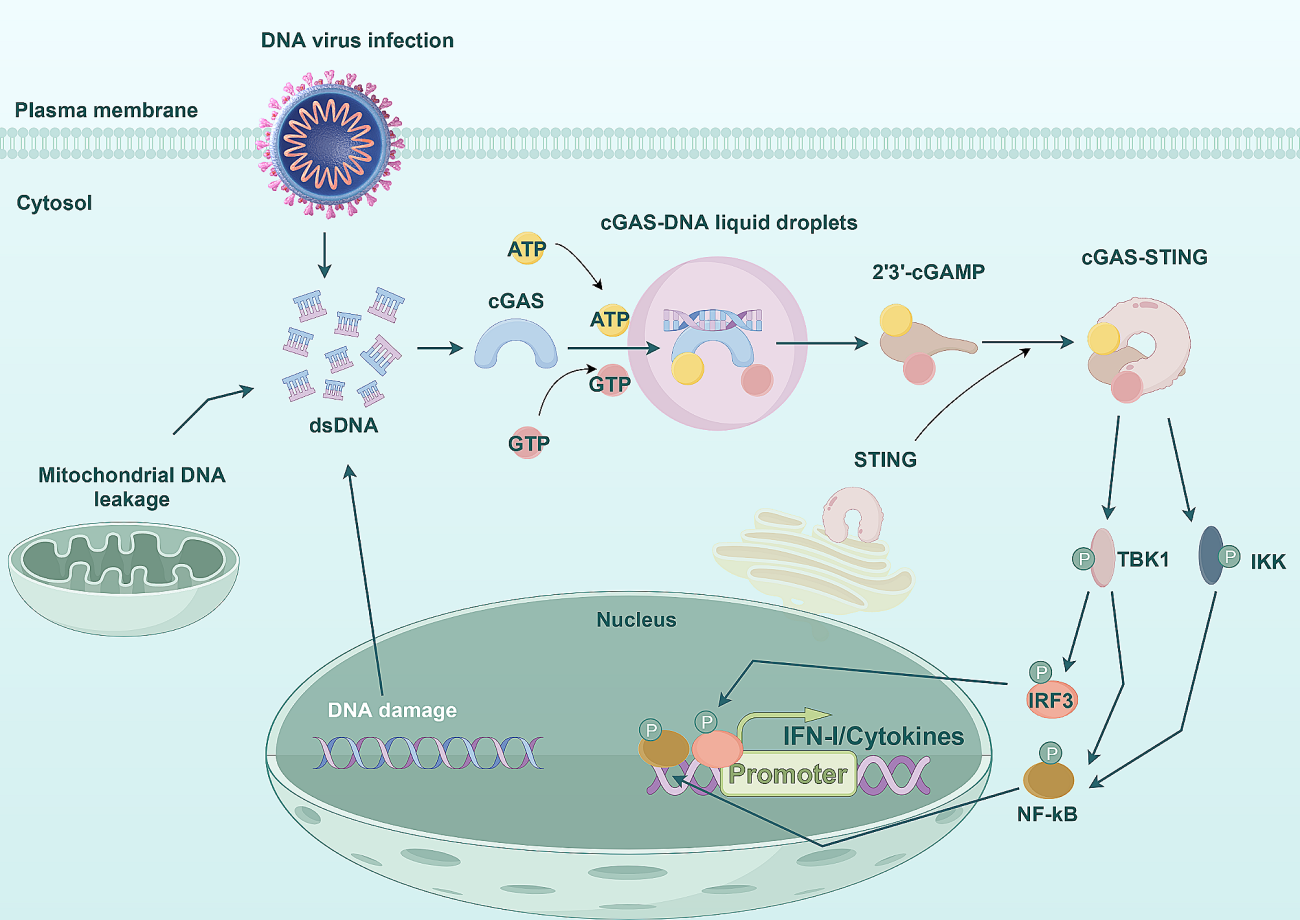
cGAS can recognize cytoplasmic dsDNA in cancer cells caused by DNA virus infection, genomic DNA damage or mitochondrial DNA leakage. Subsequently, cGAMP can be catalyzed by cGAS using ATP and GTP as substrates, which can activate and stimulate STING, leading to alteration in its conformation and activation of TBK1 and IKK. This results in the activation and phosphorylation of IRF3 and NF-κB, and phosphorylation promotes the nuclear translocation of IRF3 and NF-κB, which induces the expression of IFN I and other cytokines related to immune regulation

STING is a protein localized in the endoplasmic reticulum, and cGAS (cyclic GMP-AMP (cGAMP)) synthase serves as a cytoplasmic double-strand DNA (dsDNA) sensor. Previous studies have demonstrated that cytoplasmic dsDNA in cancer cells caused by DNA virus infection, genomic DNA damage or mitochondrial DNA leakage can be recognized by cGAS, which can activate cGAS. Subsequently, cGAMP can be catalyzed by cGAS using adenosine triphosphate (ATP) and guanosine triphosphate (GTP) as substrates, which can bind to STING and activate STING to form the cGAS-STING pathway [37, 38]. Activation of STING alters its conformation and activates TANK-binding kinase 1 (TBK1) and IκB kinase (IKK), resulting in activation and phosphorylation of interferon regulatory factor 3 (IRF3) and nuclear factor κB (NF-κB). Furthermore, phosphorylation promotes the nuclear translocation of IRF3 and NF-κB, which induces the expression of IFN I and other cytokines related to immune regulation, ultimately inducing the innate antiviral response (Fig. 2) [38, 39]. In addition to its antiviral role in innate immunity, the cGAS-STING pathway is increasingly recognized as a pivotal player in regulating tumor development [37].

Currently, the cGAS-STING pathway has demonstrated great potential in overcoming drug resistance and enhancing anti-tumor immunity, which can be activated by STING agonists or drugs related to the activation pathway, thus playing an important role in immunotherapy. When ICB therapy is employed in patients with solid tumors, responders are characterized by a ‘hot’ phenotype defined by T lymphocyte infiltration, whereas non-responders may be characterized by a ‘cold’ phenotype marked by T cell loss or exclusion in the tumor parenchyma [9, 40]. It is worth mentioning that the cGAS-STING pathway is a key pathway for activating NKs to impede the growth of T cell drug-resistant (cold) tumors [41, 42]. Augmented STING in tumor cells reprograms the TME, rendering ‘cold ' tumors sensitive to checkpoint inhibitors, thus transforming ‘cold’ tumors into hot’ tumors, which enhances the efficacy of anti-tumor immunotherapy [43].

In summary, STING is a critical signal transduction molecule that facilitates the in vivo innate immune response. High expression of STING inhibits the reactivation and proliferation of dormant NSCLC tumor cells, thereby improving the overall survival rate of patients. More importantly, STING can be activated by cGAS through the second messenger cGAMP of cyclic dinucleotide (CDN) [18].The established cGAS-STING pathway plays an important role in connecting anti-cancer innate immunity and adaptive immunity [44], thereby offering a novel strategy for the treatment of NSCLC.

## The regulation of STING activation by various factors in NSCLC

### The activation of STING can be stimulated by diverse factors

#### Ionizing radiation stimulates STING activation in radiotherapy

Presently, it is acknowledged that the STING pathway has great potential to improve anti-tumor immunotherapy. Consequently, current research will concentrate on determining how to activate the STING pathway. Previous studies have shown that activation of the cGAS-STING pathway mediated by cytoplasmic DNA sensing is associated with the triggering of an immune response during radiotherapy for NSCLC. Ionizing radiation induces DNA double-strand breaks (DSBs) during radiotherapy for NSCLC, and the DNA fragments permeate damaged nuclear membranes, enhancing the accumulation of double-stranded DNA (dsDNA) in the cytoplasm. Subsequently, cGAS stimulates the activation of the cGAS-STING pathway by sensing the accumulation of dsDNA, thereby enhancing anti-tumor immunity [45]. Additionally, DNA damage induced by targeting the ribonucleotide reductase regulatory subunit M2(RRM2) can enhance the activation of the cGAS-STING signaling pathway. The silencing of RRM2 can increase CD8^+^ T cell infiltration, which can synergistically inhibit LUAD cell proliferation and promote apoptosis with radiotherapy [46].

#### DNA damage response inhibitor (DDRI) stimulates STING activation

Recent studies have revealed that DNA damage response inhibitors (DDRI), such as poly ADP-ribose polymerase inhibitors (PARPI), can enhance anti-tumor immunity by activating the cGAS-STING pathway [47]. Research has validated that PARPI can selectively target cancer cells with DNA repair defects, including those resulting from BRCA1 mutations or excision repair cross-complementing group 1 (ERCC1) defects [48]. Notably, the ERCC1 defect is the most common DDR defect in NSCLC, with an incidence ranging from 30 to 50% [49]. PARPi, such as Olaparib and Rucaparib, can exhibit the characteristics of cell-autonomous immune regulation in ERCC1-deficient NSCLC cells. By triggering cGAS-STING signal transduction, PARPi induces downstream IFN-I signal transduction and lymphatic attractant chemokines such as CCL5 secretion, which increases the expression of PD-L1 on the surface of tumor cells induced by IFN-γ [48]. Consequently, this fosters a conducive environment for ICI therapy. In addition to its potential involvement in ERCC1-deficient NSCLC, PARPI can inhibit tumor growth in NSCLC with epidermal growth factor receptor (EGFR) mutations. Currently, EGFR mutations are detected in 51.4% of NSCLC patients in Asia [50]. Most patients are treated with EGFR-tyrosine kinase inhibitor (TKI) targeted therapy or PD-1/PD-L1 inhibitor immunotherapy, but the therapeutic efficacy is suboptimal [51, 52]. Reports indicate that PARPI (Niraparib) serves not only as a potential radiosensitizer to amplify unrepaired DNA damage but also augments the effect of radiotherapy (RT) with reduced toxicity [53], thereby impeding the growth of EGFR-mutated NSCLC tumors. Simultaneously, the synergistic effect of RT combined with niraparib fosters an anti-tumor immune response by augmenting CD8^+^ T lymphocyte infiltration and activating the STING-TBK1-IRF3 pathway [54].

Moreover, PARPI plays an important role in the treatment of SCLC. SCLC, an aggressive subtype of lung cancer, has a non-inflammatory TME and minimal cytotoxic T-cell infiltration, rendering it resistant to single or dual-drug treatment [55]. Therefore, the treatment for SCLC has limitations that lead to poor prognosis. It is essential to develop a new strategy to overcome its resistance to treatment. Currently, the combination of PARPI (Niraparib) and radiotherapy can amplify the efficacy of anti-PD-1 immunotherapy for SCLC. This synergy involves activating the cGAS-STING immune response pathway, inducing immunogenic cell death, and elevating PD-L1 expression on tumor cells to reshape the inflammatory tumor microenvironment [56]. In summary, PARPI, which is classified as a DDRI, plays a pivotal role in treating lung cancer by activating the STING pathway. Although defects in DDR can induce genomic instability in cells and contribute to the initiation and progression of cancer via mutation accumulation, they also provide targeted vulnerabilities that are relatively specific to cancer cells, thus DDRI can be utilized to achieve clinical benefits in these instances [57].

#### STING agonists stimulate STING activation

STING agonists play an important role in the immunotherapy of NSCLC. When combined with PARRI to treat NSCLC with Kelch-like ECH-associated protein 1 (KEAP1) mutation, they can also function as an adjuvant for tumor vaccines in anti-tumor therapy [58]. To achieve an optimal effect, STING agonists must efficiently target the intrinsic STING of cancer cells, eliminate disseminated cancer cells, and inhibit recurrence during the inert stage of NSCLC metastasis, which involves not only STING in NSCLC cancer cells but also NK, CD4^+^ T, and CD8^+^ T cells [34].

5,6-Dimethylxanthenone-4-acetic acid (DMXAA), a flavonoid with putative anti-tumor activity, was originally developed as an anti-angiogenic agent and was later found to interact directly with STING [59]. Although phase III clinical trials did not demonstrate any improvement in the prognosis of NSCLC patients treated with DMXAA in combination with standard chemotherapy [60], researchers have revealed that the expression levels of TNF-α, IFN-β, and STING were significantly up-regulated in EML4-ALK NSCLC tumors after DMXAA treatment, which resulted in the inhibition of tumor growth and the induction of strong anti-tumor immunity in EML4-ALK NSCLC mouse models [61]. EML4-ALK is a unique molecular subgroup of lung cancer that is resistant to checkpoint inhibitors [62]. However, investigating STING agonists in NSCLC provides a potentially beneficial avenue for the development of novel treatment strategies in addition to ICIs. Moreover, a significant proportion of STING agonists are natural CDNs derived from bacterial or human sources that function by mimicking the natural ligand 2’ 3’-cGAMP of STING, such as c-di-GMP and c-di-AMP et al. [63]. Additionally, clinical trials are currently underway for some synthetic CDN and non-CDN STING agonists, such as ADU-S100, MK-1454, and others [64]. At present, the therapeutic application of STING agonists in tumors can be specifically targeted to specific cancer cells by intravenous injection of nanoparticles carrying STING agonists, which can cause a systemic immune response, as opposed to injecting STING agonists into specific cancer cells to induce a local immune response. The TME is thereby transformed from a ‘cold’ into a ‘hot’ state that is conducive to an anti-tumor immune response. Furthermore, previous studies have reported that the STING agonist (c-di-GMP) can be encapsulated by lipid nanoparticles (LNP), resulting in the formation of STING-LNP, which was successfully delivered to the cytoplasm, thereby successful induction of anti-tumor immunity through the activation of CD8^+^ T and NK cells [65, 66]. Additionally, it is worth mentioning that before STING targeted therapies can be applied in the clinic, a thorough and comprehensive evaluation of the impact of different tumor stages, tumor microenvironment and basal cGAS-STING levels on response to STING agonist therapy is needed to determine the patients who will benefit from STING agonist therapy and we need do more research on this field [67].

#### Glycolysis stimulates STING activation in DCs

A certain correlation has been observed in the realm of cancer research between alterations in cell activation and changes in cellular metabolic state [68]. By changing the metabolic level of cancer cells, tumor growth may be affected. Likewise, altering the metabolic state of immune cells potentially impacts the anti-tumor immune response. Dendritic cells (DCs) hold a pivotal position in the immune system, as they recognize danger signals by specifically expressing a set of pattern recognition receptors (PRRs), which play a role in initiating the activation of antigen-specific T cells in both innate and adaptive immunity [69]. Alteration in metabolism is intricately linked to the successful activation of DCs. By changing the metabolic state of DCs, the inflammatory and immune responses can be modified [70]. At the metabolic level, the activation of DCs is closely related to glycolysis [71]. Research has indicated that enhanced glycolytic efficiency in tissue samples obtained from NSCLC patients increased the production of glycolytic ATP in tumor-infiltrating DCs, thereby activating STING signal transduction to promote DC-mediated anti-tumor immune response. Conversely, endogenous STING activation in DCs can also promote glycolysis and establish a positive feedback loop [72]. Therefore, glycolysis can stimulate STING in NSCLC patients to enhance the anti-tumor immune effect of DCs. Moreover, it has been observed that activated STING can boost the efficiency of glycolysis, indicating that the activation of STING is associated with the metabolic state of cells. In conclusion, STING in DCs driven by glycolysis holds great importance for anti-tumor immunotherapy in NSCLC.

### The activation of STING can be inhibited by diverse factors

#### MET amplification in lung tumors inhibits STING activation

Mesenchymal epithelial transforming factor receptor (MET) is one of the carcinogenic drivers of lung cancer and a potential therapeutic target for various cancers, including NSCLC [73]. Anomalies in MET signal transduction can drive diverse cancer types via various molecular mechanisms, encompassing MET gene amplification, mutation, rearrangement, and overexpression [74]. MET amplification is a common determinant of acquired resistance to EGFR-TKI among lung cancer patients harboring EGFR mutations [75]. MET disorders are observed in up to 26% of NSCLC patients after TKI treatment [76]. Studies have shown that increased MET signal transduction leads to sustained bypass activation of carcinogenic pathways downstream, which inhibits apoptosis and promotes tumor proliferation [75]. Additionally, MET amplification in lung tumors induces UPF1 phosphorylation, thereby down-regulating STING expression in tumor cells by modulating the length of STING’s 1′ -UTR through UPF3, resulting in a reduced STING-mediated IFN response and a decrease in tumor-infiltrating CD8^+^ T and NK cells, thereby affecting the efficiency of the ICB [77].

Additionally, MET-amplified EGFR-TKI-resistant cells induce CD73, which is responsible for producing the immunosuppressive metabolite adenosine and inhibiting the activation of STING, potentially promoting tumor progression. Notably, a statistically significant correlation was observed between high CD73 expression and poor clinical outcomes of tumors [78–80]. MET amplification in EGFR-TKI-resistant NSCLC cells produced a broader spectrum of cellular state changes related to the induction of STING and CD73. The immunogenicity of MET-amplified EGFR-TKI-resistant cells is enhanced, and STING induction and T-cell reactivity in tumor cells are promoted by inhibiting CD73 [79]. The recent assessment of the phase II COAST clinical trial, which investigated the combination of the CD73 inhibitor oleclumab and durvalumab (anti-PD L1) for treating locally advanced NSCLC, has demonstrated potential in prolonging the overall survival of patients after radiotherapy and chemotherapy [81].This outcome is strongly linked to the activation of the STING pathway. Therefore, CD73 emerges as a novel target for the treatment of NSCLC with EGFR mutation and inhibiting CD73 to promote STING induction in tumor cells offers a new strategy for NSCLC treatment. In summary, MET amplification in NSCLC cells and CD73 expression in MET-amplified EGFR-TKI-resistant cells can significantly reduce STING levels and anti-tumor T cell infiltration, thereby compromising the efficacy of ICB therapy for NSCLC.

#### Transcription factor NRF2 inhibits STING activation

The transcription factor NRF2 is a key regulator of the inflammatory response. NRF2 remains inactive in the cytosol at a steady state due to the inhibitory effect of KEAP 1, which targets NRF2 for proteasome degradation [82]. KRAS mutation, a common driver of lung adenocarcinoma, is associated with KEAP1 loss-of-function mutations in approximately 20% of KRAS mutant NSCLC cases, forming a KRAS-KEAP1 co-mutation (KK) type. KEAP1 inactivation mutations are associated with alterations in the NSCLC immune microenvironment, and the KK type shows a poor response to immune checkpoint blockade (ICB) therapy [83, 84]. When KEAP1 is inactivated under oxidative stress, NRF2 is released to induce transcription of NRF2 response genes. However, NRF2 has recently been identified as a negative regulator of STING expression because of its impact on the stability of STING mRNA [85]. Moreover, it has been demonstrated that elevated levels of NRF2 promote cancer cell proliferation in numerous types of cancer cells, including lung cancer. Constitutively high levels of NRF2 can protect cancer cells from ionizing radiation and confer radioresistance in NSCLC cells, thereby impeding radiotherapy for NSCLC [86, 87]. Additionally, DNA damage is a direct activator of the STING pathway, and BRCA1 is presently recognized as an important DNA damage repair gene. Mechanistically, NRF2 repairs DNA damage by promoting the transcription and expression of BRCA1. It can lead to the inactivation of the STING pathway [83], which is not conducive to KK-type ICB therapy. Consequently, to activate the STING pathway to enhance the efficiency of ICB therapy, the application of NRF2 inhibitors or STING agonists in the ICB therapy of KK tumors will provide a new strategy for NSCLC treatment.

#### DNA methyltransferase 1 (DNMT1) inhibits STING activation

DNA methyltransferase 1 (DNMT1), together with DNMT2, DNMT3A, DNMT3B, and DNMT3L, are all members of the DNA methyltransferase family, which regulates target gene expression through CpG island methylation [88]. Research has shown that STING is highly expressed in cancer tissues, and DNMT1 can mediate STING inhibition by interacting with nuclear paraspeckle assembly transcript 1 (NEAT1). This regulation impacts the infiltration of cytotoxic T cells in lung cancer by suppressing the cGAS-STING pathway [89, 90]. The ineffectiveness of ICB therapy in treating KRAS-LKB1 (KL) mutant lung cancer can be partly attributed to the lack of PD-L1 expression in KL-type tumors [91]. Additionally, LKB1 deletion significantly silences STING expression, thereby rendering it insensitive to dsDNA induction [89]. Mechanistically, LKB1 inactivation results in increased serine utilization and S-adenosylmethionine (SAM) synthesis [92]. Elevated SAM levels are caused by the overactivation of substrates for various epigenetic silencing enzymes, such as DNMT1 and EZH2. Studies suggest that the overactivation of DNMT1 and EZH2 can suppress STING expression in KL cells. Consequently, the loss of LKB1 in KL tumors may be related to DNMT1, leading to the poor efficacy of immunotherapy targeting KL tumors [89]. In summary, DNMT1 interacts with NEAT1 to occupy the STING promoter region and inhibit STING expression in lung cancer [90]. It also cooperates with EZH2 to control STING silencing in KL-type tumors [93]. Therefore, targeting DNMT1/EZH2 with inhibitors to induce epigenetic reprogramming and restore STING expression holds significant promise for enhancing the efficacy of ICB therapy in NSCLC [89].

#### SKIL overexpression inhibits STING activation

SKIL, also known as SnoN, functions as a mediator in the transforming growth factor-β (TGF-β) signaling pathway. Initially, it served as an oncogene by inhibiting the TGF-β/Smad pathway, thereby promoting cancer [94]. The relative expression level of SKIL in tumor tissues of NSCLC patients exhibited a positive correlation with the expression level of TAZ, a transcriptional regulator. Previous studies have shown that increased expression levels of SKIL in NSCLC facilitate autophagy by up-regulating TAZ and fostering tumorigenesis and immune escape via inhibition of the downstream STING pathway [95]. Conversely, silencing SKIL increases the expression level of STING and phosphorylation of downstream signaling pathway factors, such as TBK1 and IRF3 [96]. This indicates that SKIL silencing may affect T cell infiltration and the release of related chemokines by activating the STING pathway [95]. By inhibiting SKIL expression in cancer cells, the adaptive immunity of tumor cells can be activated, thereby enhancing the efficacy of anti-tumor immunotherapy and introducing a novel treatment strategy for NSCLC.

## The latest research progress of STING in NSCLC treatment

### STING functions in chemotherapy and immunotherapy for NSCLC

Before the era of immunotherapy, the first line of therapy for NSCLC was platinum-based chemotherapy [97]. For patients with advanced NSCLC, standard chemotherapy included the combination of platinum (carboplatin or cisplatin) with gemcitabine, vinorelbine, or taxane such as paclitaxel or docetaxel [98]. Since the advent of the immunotherapy era, studies have indicated that patients who integrate standard chemotherapy (platinum-based double drugs) with immunotherapy as their first-line therapy have a higher overall survival rate than those who receive chemotherapy alone [99]. Both carboplatin and cisplatin can activate the STING pathway in NSCLC and enhance the anti-tumor activity of PD-1 inhibitors. Moreover, low-dose carboplatin can induce DNA damage and activate the canonical STING-TBK1-IRF3 pathway and non-canonical STING-NF-κB signaling complex. Platinum chemotherapy is beneficial for transforming ‘cold’ tumors into ‘hot’ ones, enhancing CD8^+^ T cell infiltration and increasing PD-L1 expression. Thus, it increases the anti-tumor effect of PD-1 inhibitors [11, 100]. However, STING deletion in cancer cells can effectively reverse the up-regulation of PD-L1 and activation of the STING pathway, consequently attenuating the anti-tumor effect of low-dose carboplatin in combination with carboplatin-PD-1 inhibitor [100]. This suggests that the enhancement of the anti-tumor immune response by carboplatin can be mediated by the STING pathway.

In addition to cisplatin and carboplatin, pemetrexed (PEM), a multi-target folate antagonist, is commonly combined with cisplatin in the chemotherapy of NSCLC patients. Compared with the effect of cisplatin and gemcitabine in treating all NSCLC patients, the pemetrexed combination was observed to be better tolerated, and it is the most commonly used cytotoxic drug in EGFR-mutated lung cancer [98, 101]. Yu et al. found that that pemetrexed or cisplatin alone can cause the activation of the STING-TANK-TBK1-IRF3 pathway and the STING-ataxia telangiectasia mutated (ATM)-IFI16-NF-κB pathway in tumor tissues. They indicated that sequential treatment with pemetrexed and cisplatin induces activation of the STING pathway both in vivo and in vitro settings in NSCLC patients. This activation enhances the infiltration of CD8^+^ T cells and up-regulates the level of PD-L1, which provides a theoretical foundation for the development of clinical medication [102]. In the case of EGFR-TKI treatment failure, the combination of PEM and CD73 inhibitors can synergistically induce cancer cell STING and increase immunogenicity in TKI-resistant EGFR mutant lung cancer. Consequently, PEM has been recognized as a potent enhancer of STING-dependent TBK3-IRF1-STAT73 signal transduction in MET-amplified EGFR-TKI-resistant cells (Table 1) [79].

**Table 1:** The mechanism of STING works in the treatment of NSCLC

Therapy	Medicine	Mechanism	Reference
Chemotherapy	Carboplatin	STING-TBK1-IRF3	[100]
		STING-NF-κB	[100]
	Cisplatin	STING-TANK-TBK1-IRF3	[102]
		STING-ATM-IFI16-NF-κB	[102]
	PEM	STING-TANK-TBK1-IRF3	[102]
		STING-ATM-IFI16-NF-κB	[102]
		STING-TBK3-IRF1-STAT73	[79]
RT	/	cGAS-STING	[45]
MTT	Anlotinib	cGAS-STING-IRF3	[104]
	Osimertinib	cGAS-STING	[105]
NT	MDPMH	cGAS-STING	[117]
	Bio-MnO2 NPs	cGAS-STING	[118]
	IO-PG-GLU-Ce6	cGAS-STING	[119]

Abbreviations: PEM pemetrexed, RT radiotherapy, MTT molecular targeted therapy, NT nano-technology, MDPMH Mn-modified phthalocyanine derivative @ docetaxel @ PLGA @ Mn2^+^ @ HA, Bio-MnO2 NPs biomineralized manganese oxide nanoparticles, IO-PG-GLU-Ce6 iron oxide-polyglycerol-glucose-chlorin e6

In summary, chemotherapy induces PD-L1 up-regulation by activating the STING pathway, which can alter the local immune status and optimize the administration of immunosuppressants, thereby enhancing the anti-tumor immune effect of NSCLC. Thus, STING plays an important role in NSCLC chemotherapy and immunotherapy.

### STING plays a role in radiotherapy and molecular targeted therapy for NSCLC

RT is commonly used to antagonize both primary and metastatic tumors. In patients with stable disease and fewer than three metastases, consolidation radiotherapy for metastatic sites has demonstrated a superior overall survival rate compared to relying solely on maintenance systemic therapy [98]. The tumor-killing effect induced by RT can stimulate T cells to release IFN-I and produce a pro-inflammatory environment. Moreover, ionizing radiation (IR) can induce DSB, thereby directly killing cancer cells [103, 104]. Additionally, IR enhances the anti-tumor immune effect by elevating the accumulation of cytoplasmic dsDNA, which can be sensed by cGAS, leading to the formation of the cGAS-STING pathway [45]. According to research, the combination of PD-L1 inhibitor and IR may amplify IR-induced cGAS-STING signal transduction and stimulate the response of T cell activation. Moreover, this combined approach effectively controls the abnormal enlargement of the spleen and T lymphocyte injury induced by tumor growth, indicating that IR combined with PD-L1 inhibitors also plays an important role in maintaining spleen function during the treatment of NSCLC [103].

Furthermore, previous studies have revealed that anlotinib, a third-generation NSCLC targeted drug, serves as a small molecule multi-target tyrosine kinase inhibitor, which exhibits a significant effect on radioimmunotherapy. Han et al. found that anlotinib can enhance radiosensitivity, leading to increased CD8^+^ T cells infiltration and radiotherapy activation through the activation of the cGAS-STING-IRF3 pathway. The triple therapy, including IR, PD-L1 inhibitor, and anlotinib, can alleviate the depletion of CD8^+^ T cells, augment their cytotoxicity and proliferation, and enhance immune memory activation. This collectively renders tumors more responsive to both radiotherapy and radioimmunotherapy, which ultimately improves therapeutic outcomes [104]. Beyond anlotinib, Vicencio et al. have revealed that osimertinib, the third-generation EGFR-TKI, can activate cGAS in tumor cells to produce cGAMP, promoting surrounding macrophages to increase STING-mediated type-I IFN responses [105]. Additionally, osimertinib (AZD9291) can enhance tumor T-cell infiltration when combined with MSA-2 (a small molecule non-nucleotide STING agonist suitable for systemic administration). This is achieved through reprograming tumor-associated macrophages (TAMs), which overcomes its limitation on the efficacy of osimertinib, thereby reinstating the antitumor activity of osimertinib in triggering T-cell activation and promoting long-lasting tumor regression [106]. This combination addresses the issue of osimertinib resistance observed in most patients (Table 1).

In summary, radiotherapy can stimulate the activation of the STING pathway through radiation-induced DSBs, thereby inducing the activation of innate and adaptive immune responses against tumors [107]. Combining radiotherapy with anlotinib can inhibit the repair of IR-induced DNA double-strand breaks, increase the accumulation of cytoplasmic dsDNA, and enhance the activation of the STING pathway in EGFR mutation-positive patients [104]. Moreover, the combination of the STING agonist MSA-2 and EGFR-TKI osimertinib (AZD9291) has been observed to up-regulate the production of anti-tumor cytokines (i.e., IFNγ and TNFα) in CD8^+^ and CD4^+^ T cells, thereby enhancing the efficacy of osimertinib [106]. Therefore, STING plays an important role in radiotherapy and molecular-targeted therapy for NSCLC patients.

### STING plays a role in conventional treatment with a new strategy for NSCLC

Currently, conventional therapy remains the primary treatment strategy for NSCLC, without alternative strategies such as chemotherapy, surgery, radiotherapy, or synergistic therapy. Nevertheless, patients frequently endure excruciating pain due to surgery and side effects of chemotherapy and radiotherapy, including dose dependence and low selectivity [98]. Therefore, due to the importance of selecting a drug delivery medium for NSCLC patients that specifically targets cancer cells in the lung without damaging normal tissues, nanoparticles(NPs) were developed as a drug delivery medium in combination with NSCLC treatment.

Currently, NPs is applied in the field of lung cancer treatment with a broad range. In comparison to conventional delivery methods, utilizing NPs for targeted drug delivery to the lungs can effectively overcome various pulmonary barriers and facilitate precise tissue localization, thereby enhancing therapeutic efficacy while minimizing systemic side effects [108]. Therefore, it is essential to choose a proper way to deliver NPs to cancer cells. The administration of NPs can be achieved through various routes, such as intravenous/intraperitoneal injection, oral ingestion, and pulmonary inhalation. Among these, the most commonly employed route is intravenous injection [109]. Abraxane, the albumin-bound paclitaxel nanoparticle delivered to lung by intravenous injection, is the most well-known nano-formulated drug for NSCLC, which shows significant retardation of tumor growth [110]. Besides chemotherapy drugs, NPs with immunomodulatory functions can trigger immune cells and adjust TME to improve antitumor immunity [111]. Recently, Reda et al. have designed a nanoparticle-based immunotherapy system called Antigen Release Agent and Checkpoint Inhibitor (ARAC) in order to improve the PD-L1 inhibitor’s efficiency [112]. Additionally, Ajam-Hosseini et al. mentioned that NP-encapsulated oncolytic viruses (OV) complex promises increased tumor tissue lysis with reduced side effects [113]. Yang et al. found that OVs can reverse the immunosuppressive microenvironment and promote the maturation of immune cells by turning a cold tumor into a hot one and induce the activation of the cGAS-STING pathway [114]. This activation causes the production of cytokines and type I IFNs, which leads to the maturation of immune cells such as DC and NK cells that can enhance anti-tumor effect [113]. Consequently, it is reasonable to speculate that STING pathway can play a vital role in the conventional treatment with NPs.

Previous studies have indicated that Mn^2+^ can activate the STING pathway. It has the potential to increase the sensitivity of cGAS to cytoplasmic DNA, as well as enhance the binding affinity of cGAP and the STING protein [115]. The sustained release of Mn2 ^+^ can effectively activate the STING pathway, resulting in potent tumor-killing capabilities, which are also involved in the radiotherapy of cancers [116]. However, the targeting issue remains evident when using free Mn^2+^. It is possible to address this concern by employing nanoparticles as a delivery medium to ensure targeted delivery of Mn^2+^ and improve biosafety. For instance, Mn^III^PC@DTX@PLGA@Mn^2+^@HA (MDPMH) nanoparticles consist of novel photothermal agents, Mn-modified phthalocyanine derivative (Mn^III^PC), docetaxel (DTX), and an effective targeting molecule, hyaluronic acid. The nanoplatform could release Mn^2+^ from MDPMH and potentially activate tumor immunity through cGAS-STING and chemotherapy. This can be used for the treatment of NSCLC patients [117]. Additionally, Liu et al. reported that the biomineralized manganese oxide nanoparticles (Bio-MnO_2_ NPs) prepared by mild enzymatic reaction could be a new strategy to synergistically enhance RT and RT-induced immune responses. Bio-MnO_2_ NPs could relieve tumor hypoxia in order to augment the radiosensitivity of NSCLC cells. Meanwhile, the release of Mn^2+^ into the TME significantly enhanced the cGAS-STING activity to activate radio-immune responses, boosting immunogenic cell death and increasing cytotoxic T cell infiltration, which provides a novel strategy to induce immune responses and achieve tumor-specific therapy for NSCLC [118].

Besides Mn2 ^+^ applied in nanoplatform, iron oxide (IO) NPs have been widely used in clinics recently. Yu et al. have prepared IO–polyglycerol (PG)– glucose (GLU)–Chlorin e6 (Ce6) composites, and they demonstrated that the accumulation of Ce6 produced more reactive oxygen species (ROS), thereby inhibiting the viability, proliferation, and promoting the apoptosis of LLC. Furthermore, compared with free Ce6, the composites damaged DNA, activated molecule of STING, up-regulated the expression of IFN-β, HMGB1, HSP90, thus effectively enhancing immunogenicity that provides a promising strategy for treating NSCLC (Table 1) [119].

In summary, the combination of nanoparticles with STING to enhance anti-tumor immunity is a highly regarded area of research. Currently, various new combinations have been developed. Some of these involve the combination of nanoparticles with STING agonists, while others involve a combination with Mn^2+^, and both of these can assist in the activation of the cGAS-STING pathway in cancer cells. However, although nanotechnology has broad application prospects, it also has faced kinds of challenges. For example, it is more difficult to control the physicochemical properties of these smart nano-formulations due to their complexity and factors such as disintegration and/or biodegradation of NPs, leakage of loaded drugs, and dissociation of surface-modified components can affect the stability of NP delivery systems [120, 121]. In addition, certain studies have shown that the accumulation of nanodrugs in healthy organs and tissues can lead to systemic side effects [122]. Another challenge is how to combine the function of STING with NPs and active the STING pathway in lung cancer cells. Currently, the clinical translation of STING agonists is constrained by several factors, such as the complexity of STING structure, poor serum stability of agonists, widespread STING expression, and pleiotropy of STING signaling [67]. The clinical trials about STING with NPs implemented in NSCLC are still not enough. Nakamura et al. observed that in lung metastasis of a B16-F10 mouse melanoma, STING agonist loaded LNPs can efficiently induced antitumor activity via the activation of NK cells, which proved that STING-LNP can overcome anti-PD-1 resistance in melanoma lung metastasis [123]. Similar to this study, we need do more research about STING-LNP applied in lung cancer cells to prove its efficiency in clinic. Furthermore, the challenge about choosing the receptor that only exists in the lung through elaborate studies to further improve the effect of lung-targeted drug delivery and how to deliver NPs to NSCLC cells need to make more researches [108].

## Concluding remarks and future perspectives

STING serves as a pivotal signal transduction molecule in the in vivo innate immune response and is activated by exogenous DNA. This plays a crucial role in regulating the process of the in vivo spontaneous anti-tumor immune response. When activated, STING can inhibit the proliferation and metastasis of NSCLC cancer cells. However, STING exhibits a dual function, promoting tumor progression in specific situations, such stimuation by tumor antigenicity and certain enzymes. Currently, the activation of STING is primarily observed to be beneficial in inhibiting tumor progression in various NSCLC treatments. The activation of the STING pathway penetrates various programs in NSCLC, including chemotherapy, radiotherapy, molecular targeted therapies, immunotherapy, and others, to enhance the anti-tumor immune effect. At present, the construction of nanoparticle combinations for delivering STING agonists or Mn^2+^ in conjunction with certain anti-cancer drugs achieves targeted activation of the STING pathway at specific tumor sites. This approach ensures the targeting effect and biosafety of the delivered drug in vivo.

It is worth mentioning that the use of messenger RNA (mRNA) for vaccination, protein replacement therapy and cancer immunotherapy holds the potential to revolutionize the treatment of a wide range of diseases [124]. In recent years, researchers pay more attention on searching new therapy like combining NPs with mRNA vaccines for targeted delivery to improve the efficacy of these vaccines. They found the best candidates for vaccine mRNA transfer are LNPs, which are stable and biocompatible [125]. Additionally, Qiu et al. have developed a library of N-series LNPs that could specifically regulate the protein composition of protein corona on the surface of LNPs, which allows specific delivery of mRNA to the lung. They demonstrated that nLNPs could effectively deliver mouse tuberous sclerosis complex 2 (Tsc2) mRNA into TSC2-null cells to treat pulmonary lymphangioleiomyomatosis [124]. Based on these findings, we put forward an outlook that it can be considered to combine STING mRNA with specific lung-targeted NPs such as nLNPs to effectively deliver STING mRNA to the lung, and through activating STING to inhibit tumor proliferation and metastasis, which add a feasible dimension to NSCLC therapy. Although, there are some challenges facing STING mRNA-nLNPs like the poor stability of mRNAs and the information on the structure and morphology of mRNA-formulated LNPs is limited. Furthermore, the effect of mRNA encapsulation in LNPs on the stability of STING mRNA storage is still unknown. We believe through more researches on the role of STING in the treatment of NSCLC and the remarkable progress of nanotechnology, the clinical efficacy for NSCLC patients will be significantly improved, which provide multi-dimensional treatment for NSCLC in future.

## Data Availability

No datasets were generated or analysed during the current study.

## Abbreviations

STING: Stimulator of interferon genes
NSCLC: Non-small cell lung cancer
SCLC: Small cell lung cancer
ICB: Immune checkpoint blockade
CTLA-4: Cytotoxic T lymphocyte antigen 4
PD-1: Programmed cell death receptor 1
PD-L1: Ligand programmed cell death ligand 1
TME: Tumor microenvironment
DMBA: Polyaromatic hydrocarbon 7,12-dimethylbenz[α]anthracene
IDO: Indoleamine 2,3-dioxygenase
LLC: Lewis lung cancer
TDLNs: Tumor-draining lymph nodes
IFNAR: IFN-I receptor
MDSC: Myeloid-derived suppressor cell
Treg: Regulatory T cells
SAMHD1: Sterile alpha motif domain and histidine-aspartate domain-containing protein 1
dNTP: Deoxynucleotide triphosphate
ssDNA: Single-stranded DNA
IFI 16: IFN-γ-inducible protein 16
LUAD: Lung adenocarcinoma
IFNβ: Interferon-β
cGAMP: Cyclic GMP-AMP
dsDNA: Cytoplasmic Double-Strand DNA
TBK1: TANK-binding kinase 1
IKK: IκB kinase
IRF3: Interferon regulatory factor 3
NF-κB: Nuclear factor κB
CDN: Cyclic dinucleotide
DSBs: DNA double-strand breaks
RRM2: Ribonucleotide reductase regulatory subunit M2
DDRI: DNA damage response inhibitor
PARPI: Poly ADP-ribose polymerase inhibitors
ERCC1: Excision repair cross complementing group 1
EGFR: Epidermal growth factor receptor
TKI: EGFR-tyrosine kinase inhibitor
RT: Radiotherapy
DMXAA: 5,6-Dimethylxanthenone-4-acetic acid
LNP: Lipid nanoparticles
DCs: Dendritic cells
PRRs: Pattern recognition receptors
MET: Mesenchymal epithelial transforming factor receptor
KEAP 1: Kelch-like ECH-associated protein 1
KK: KRAS-KEAP1
DNMT1: DNA methyltransferase 1
NEAT1: Nuclear paraspeckle assembly transcript 1
KL: KRAS-LKB1
SAM: S-adenosylmethionine
TGF-β: Transforming growth factor-β
ATM: Ataxia telangiectasia mutated
IR: Ionizing radiation
TAM: Tumor-associated macrophages
NPs: Nanoparticles
ARAC: Antigen release agent and checkpoint inhibitor
OV: Oncolytic viruses
mRNA: Messenger RNA
Tsc2: Tuberous sclerosis complex 2
